# The identification of the key residues E829 and R845 involved in transient receptor potential melastatin 2 channel gating

**DOI:** 10.3389/fnagi.2022.1033434

**Published:** 2022-10-24

**Authors:** Yuhuan Luo, Shijia Chen, Fei Wu, Chunming Jiang, Marong Fang

**Affiliations:** ^1^Department of Pediatrics, Affiliated Hangzhou First People’s Hospital, Zhejiang University School of Medicine, Hangzhou, China; ^2^National Clinical Research Center for Child Health, Children's Hospital of Zhejiang University School of Medicine, Hangzhou, China; ^3^Institute of Systemic Medicine, Zhejiang University School of Medicine, Hangzhou, China; ^4^Institute of Neuroscience, Zhejiang University School of Medicine, Hangzhou, China

**Keywords:** TRPM2, key residues, gating mechanism, activation, inactivation, transmembrane segments

## Abstract

Transient receptor potential melastatin 2 (TRPM2), a non-selective cation channel, is involved in many physiological and pathological processes, including temperature sensing, synaptic plasticity regulation, and neurodegenerative diseases. However, the gating mechanism of TRPM2 channel is complex, which hinders its functional research. With the discovery of the Ca^2+^ binding site in the S2–S3 domain of TRPM2 channel, more and more attention has been drawn to the role of the transmembrane segments in channel gating. In this study, we focused on the D820-F867 segment around the S2 domain, and identified the key residues on it. Functional assays of the deletion mutants displayed that the deletions of D820-W835 and L836-P851 destroyed channel function totally, indicating the importance of these two segments. Sequence alignments on them found three polar and charged residues with high conservation (D820, E829, and R845). D820A, E829A, and R845A which removed the charge and the side chain of the residues were tested by 500 μM adenosine diphosphate-ribose (ADPR) or 50 mM Ca^2+^. E829A and R845A affected the characteristic of channel currents, while D820A behaved similarly to WT, indicating the participations of E829 and R845 in channel gating. The charge reversing mutants, E829K and R845D were then constructed and the electrophysiological tests showed that E829A and E829K made the channel lose function. Interestingly, R845A and R845D exhibited an inactivation process when using 500 μM ADPR, but activated normally by 50 mM Ca^2+^. Our data suggested that the negative charge at E829 took a vital part in channel activation, and R845 increased the stability of the Ca^2+^ combination in S2-S3 domain, thus guaranteeing the opening of TRPM2 channel. In summary, our identification of the key residues E829 and R845 in the transmembrane segments of TRPM2. By exploring the gating process of TRPM2 channel, our work helps us better understand the mechanism of TRPM2 as a potential biomarker in neurodegenerative diseases, and provides a new approach for the prediction, diagnosis, and prognosis of neurodegenerative diseases.

## Introduction

As an important non-selective cation channel, transient receptor potential melastatin 2 (TRPM2), has an endogenous expression in many organs, such as brain (Li and Jiao, 2020; Zong et al., 2022), heart (Ji et al., 2021), liver (Feng et al., 2019), kidney (Wang et al., 2019), and lung (Wang et al., 2020), and participates in various physiological and pathological processes. For example, TRPM2 channel is involved in temperature sensing (Song et al., 2016; Kamm et al., 2021), synaptic plasticity regulation (Wang et al., 2016), and insulin secretion (Ito et al., 2017). Based on its response to oxidative stress, the activation of TRPM2 channel mediates several neurodegenerative diseases, including Alzheimer’s disease (Aminzadeh et al., 2018), Parkinson’s disease (Ferreira et al., 2022), multiple sclerosis (Shao et al., 2021), and stroke (Zong et al., 2022). It is also regarded as a therapeutic target for the treatments of neurological diseases (Belrose and Jackson, 2018). However, the mechanism of TRPM2 gating is still unclear, which hinders the researches about its pathological role. Recent years have seen lots of work about TRPM2 channel structural resolution, key residues identification and gating mechanism exploration, especially its opening and closing processes (Luo et al., 2018; Wang et al., 2018; Yu et al., 2021).

Transient receptor potential melastatin 2 channel can be opened mainly by adenosine diphosphate-ribose (ADPR), Ca^2+^, and reactive oxygen species (Hu et al., 2021; Yu et al., 2021). As an efficient agonist, ADPR activates the channel through its binding in the TRPM homology region 1/2 (MHR1/2) at N-terminal of the channel, and the NUDT9 homology (NUDT9-H) domain at C-terminal (Huang et al., 2019). Although there are two binding sites for ADPR, ADPR only opens the channel in the existence of Ca^2+^ (Starkus et al., 2007), indicating a vital role of Ca^2+^ in TRPM2 activation. By the using of cryo-electron microscopy, a Ca^2+^ binding site was identified in the domain of the second and third transmembrane segment (S2-S3) of human TRPM2, composed by E843, Q846, N869, D872, and E1073 (Wang et al., 2018). What is more, this highly conserved Ca^2+^ binding site exists in various species, including human, Nematostella vectensis and zebrafish (Huang et al., 2018; Wang et al., 2018; Zhang et al., 2018), and the five residues involved also exhibit a high conservation among the TRPM family (Zhang et al., 2018). During the channel gating, Ca^2+^ can open the channel by itself (Du et al., 2009), or display a synergistic effect with ADPR, which promotes channel activation (Lange et al., 2008).

Inhibition and inactivation are the two methods for closing the channel. Former studies have discovered several compounds that can inhibit TRPM2 channel, such as N-(p-amycinnamoyl) anthranilic acid (ACA), and 2-aminoethoxydiphenyl borate (2-APB; Jiang et al., 2010; Malko and Jiang, 2020). On the other hand, some metal ions (Zn^2+^ and Cu^2+^) inactivate the channel in a concentration-dependent way. Zn^2+^ inactivates TRPM2 channel through residues K952 and D1002 (Yang et al., 2011), and Cu^2+^ inactivates TRPM2 channel through its interaction with H995 (Yu et al., 2014). Apart from all these exogenous substances, the mutations at the key residue of TRPM2 channel, also lead to inactivation. For example, the alanine and glutamate mutations at Q846, and aspartate mutation at N869 inactivated TRPM2 channel significantly (Luo et al., 2019).

Although the gating mechanism for TRPM2 channel is complex, the key amino acid residues and regions take an important part during the opening and closing of the channel. The discovery of the Ca^2+^ binding site in S2-S3 domain of TRPM2 channel drew attention to exploring the role of transmembrane segments in the channel gating process (Wang et al., 2018). Here, we focused on the S2 domain, and chose the D820-F867 segment that contains residues from the end of S1 domain to the start of S3 domain as the target segment. Apart from its close location to the Ca^2+^ binding site, D820-F867 segment is also located in the voltage-sensing-like domain (Wang et al., 2018), which increases the probability of its participation in channel gating.

In this study, we aimed to identify the key residues that involved in TRPM2 channel gating within the D820-F867 segment. Electrophysiological examination of three deletion mutants displayed the importance of D820-W835 and L836-P851 for TRPM2 gating. Sequence alignment were used to find the polar and charged residues with high conservation in these two segments. Alanine mutations and mutations reversing the charge of the residue were constructed, and their channel currents were recorded by the activation of intracellular solution containing 500 μM ADPR or 50 mM Ca^2+^. Our work identified E829 and R845 as the key residues closely related with channel gating, and expanded the understandings of the gating mechanism of the transmembrane region in TRPM2 channel.

## Materials and methods

### Cell culture and molecular biology

Human Embryonic Kidney 293 T (HEK293T) cells are widely used in the function tests of the target ion channels and its mutants because of the high transfection ability and rare expression of endogenous receptors (Wang and Wang, 2017; Glazer et al., 2020; El Ghaleb et al., 2021). In this study, HEK293T cells (purchased from ATCC) were cultured using Dulbecco’s modified Eagle medium (DMEM, Thermo Fisher Scientific, United States) with 10% fetal bovine serum (FBS; Thermo Fisher Scientific, United States) in a humidity-controlled incubator (5% CO_2_, 37°C). The cDNA encoding full-length human TRPM2 (protein accession number: NP_003298.2) was kindly provided by Dr. A. M. Scharenberg (University of Washington, Seattle, WA, United States), and was subcloned into pcDNA3.1 vector as it was reported previously (Yu et al., 2017, 2019, 2021). Mutant constructions were done through site-directed or deletional mutagenesis followed by full coding sequence sequencing (Yang and Jiang, 2013). Briefly, for each mutation: 5 μl of 10× reaction buffer, 2 μl (50–100 ng) of cDNA template, 2 μl of 10 μM sense mutagenic primer, 2 μl of 10 μM antisense mutagenic primer, and 4 μl of 2.5 mM dNTP mix were added into the PCR reaction system. The sterile DNAase-free H_2_O was then added to a final volume of 49 μl, and 1 μl of 2.5 U/μl Pfu DNA polymerase was added finally. The reaction program for the PCR: 95°C for 5 min; 20 cycles of 95°C for 30s, 58°C for 60s, and 68°C for 12 min; 68°C for 10 min. After the PCR process, 1 μl of DpnI enzyme (10 U/μl) was added to each PCR sample, and was mixed gently and thoroughly before incubated at 37°C for 1 h to remove the cDNA templates. The mutations verified by full coding sequence sequencing were used in the experiments.

### Transfection

The transfections were done following the instruction of the Lipofectamine 2000 reagent (Thermo Fisher Scientific, United States). HEK293T cells were grown in 35-mm petri dishes and transfected with 1 μg TRPM2 plasmid and 0.1 μg green fluorescent protein (GFP) plasmid (Mei et al., 2006). Briefly, 2 μl lipo2000 was added into 100 μl Opti-MEM™ I (OPTI, Thermo Fisher Scientific, United States). The solution was then mixed and stood for about 5 min. Meanwhile, 1 μg WT TRPM2 or its mutants, together with 0.1 μg GFP, were added into 100 μl OPTI, and these two solutions were mixed well and stood for 20 min. After that, the DMEM in the dishes was replaced with 800 μl fresh DMEM and the mixed solution was added into the dishes for cell culture. 24 h after the transfection, the transfected cells were seeded on glass coverslips and the electrophysiological recording were carried out 12 h later.

### Electrophysiology

Whole-cell recordings were carried out at room temperature using an Axopatch 200B amplifier. Patch electrodes with a resistance of 3–5 MΩ were fabricated from borosilicate glass (Sutter Instrument). The protocols for electrophysiological recordings were described in our previous studies (Luo et al., 2018, 2019). Briefly, voltage ramps with 500 ms duration from-100 mV to +100 mV were applied every 5 s. The currents at-80 mV were denoted by circles in figures. The intracellular solution (ICS) of 500 μM ADPR contained: 147 mM NaCl, 1 mM MgCl_2_, 0.05 mM EGTA, 10 mM Hepes, and 500 μM ADPR (pH 7.4, adjusted with NaOH). The ICS of 50 mM Ca^2+^ contained: 50 mM CaCl_2_, 75 mM NaCl, 1 mM MgCl_2_, and 10 mM Hepes (pH 7.4, adjusted with NaOH). For all experiments, the standard extracellular solution (ECS) contained: 147 mM NaCl, 2 mM KCl, 1 mM MgCl_2_, 2 mM CaCl_2_, 10 mM Hepes, and 13 mM glucose (pH 7.4, adjusted with NaOH). To confirm the TRPM2 channel currents, 20 mM N-(pamylcinnamoyl) anthranilic acid (ACA; Sigma) was applied at the end of each recording, and only the cells whose currents were completely inhibited by ACA were used for analysis.

### Statistical analysis

The electrophysiological data were analyzed using pCLAMP9 software (Axon Instruments), and presented as Mean ± SEM. Origin software was used for curve fitting. Statistical analysis was performed using Student’s *t*-test (*p* < 0.05 designated as significant).

## Results

### The deletion of D820-W835 and L836-P851 segments resulted in the loss of function of TRPM2 channel

In order to check whether D820-F867 segment participates in TRPM2 channel gating, and also to detect the possible regions where the key residues are located, we constructed three deletion mutants, that is △D820-W835 (deletion of D820-W835 residues), △L836-P851 (deletion of L836-P851 residues), △D852-F867(deletion of D852-F867 residues), and activated these three mutants by ICS of 500 μM ADPR. The inhibitor of TRPM2 channel, 20 μM ACA, was applied at the end of the experiments, to confirm the currents of TRPM2 channel.

**Figure 1:**
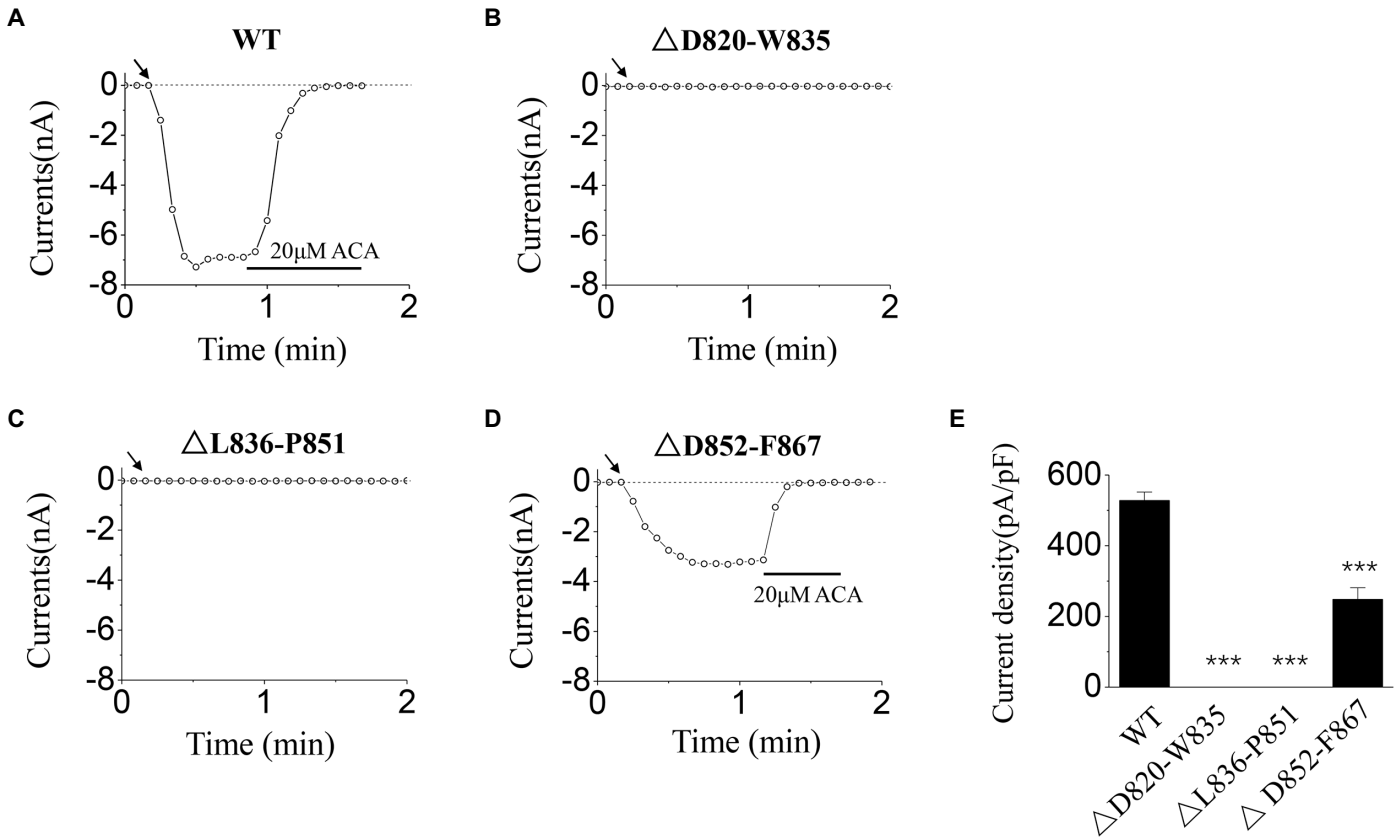
Functional assay of WT transient receptor potential melastatin 2 (TRPM2) and three deletion mutants. **(A–D)** Representative whole-cell recordings of TRPM2 channel currents induced by intracellular solution (ICS) of 500 μM adenosine diphosphate-ribose (ADPR) from Human Embryonic Kidney 293 T (HEK293T) cells expressing **(A)** WT, **(B)** △D820-W835, **(C)** △L836-P851, and **(D)** △D852-F867. The arrow in each panel indicates the time point at which whole cell configuration was established. **(E)** Summary of the current density induced in cells expressing WT and deletion mutants. In each case, six cells were tested. ^***^p < 0.001 compared with WT.

Our data showed that, △D820-W835, △L836-P851 mutants made the channel currents disappeared totally (Figures 1B,C), suggesting the existence of the key residues within them. In contrast, despite the loss of 16 residues, △D852-F867 mutant still retained part of currents of TRPM2 channel (Figure 1D), suggesting its minor role in channel gating. Based on this result, sequence alignment and electrophysiological examination were carried out for D820-W835 and L836-P851 segments in the following research.

### Sequence alignment

The high conservation is one of the characteristics of the key residues in the course of the evolution, so the sequence alignment was done to find the residues highly conserved. The D820-F867 segment of hTRPM2 (NP_003298.2) was compared with its corresponding segment in mouse TRPM2 (mTRPM2, NP_001398829.1) and mouse TRPM8 (mTRPM8, NP_599013.1), and the analysis of conservation was carried out for D820-W835 and L836-P851.

**Figure 2:**
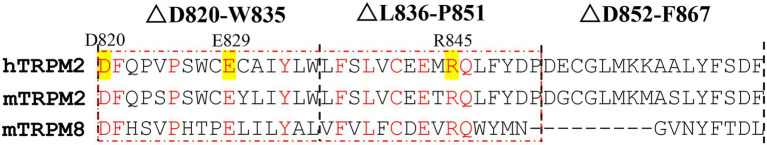
Sequence alignments between hTRPM2, mTRPM2, and mTRPM8. The sequence alignments were done for the residues in the red dashed box. Apart from the residues constitute the Ca^2+^ binding site (E843 and Q846), residues with high conservation were marked in red, and the polar and charged ones among them were highlighted in yellow (D820, E829, and R845).

As Figure 2 shows, there are 11 residues of high conservation, that is: D820, F821, P825, E829, Y833, F837, L839, C841, E843, R845, and Q846. Among them, E843 and Q846 have been reported to constitute the Ca^2+^ binding site in S2-S3 domain. According to the polarity and charge, the other nine residues were divided into three types: polar and charged residues (D820, E829, and R845; Qiao et al., 2021), polar and uncharged residues (Y833, C841), and non-polar residues (F821, F837, P825, and L839). During the channel gating, polarity and charge are two important ways for residues to interact with their surrounding residues or channel ligands. Therefore, the highly conserved residues: D820, E829, and R845, which also possess both of the polarity and charge, were most likely to participate in TRPM2 gating. In order to confirm their effects and explore the mechanism, electrophysiology of the site-directed mutants at these three residues was carried out in this study.

### Alanine mutations at E829, R845 altered TRPM2 channel currents

Here, we substituted the D820, E829, and R845 residues with alanine, which has no charge or side chain. According to our former studies (Luo et al., 2018, 2019), ICS of 500 μM ADPR and 50 mM Ca^2+^ were used in the activation tests for these mutants.

**Figure 3:**
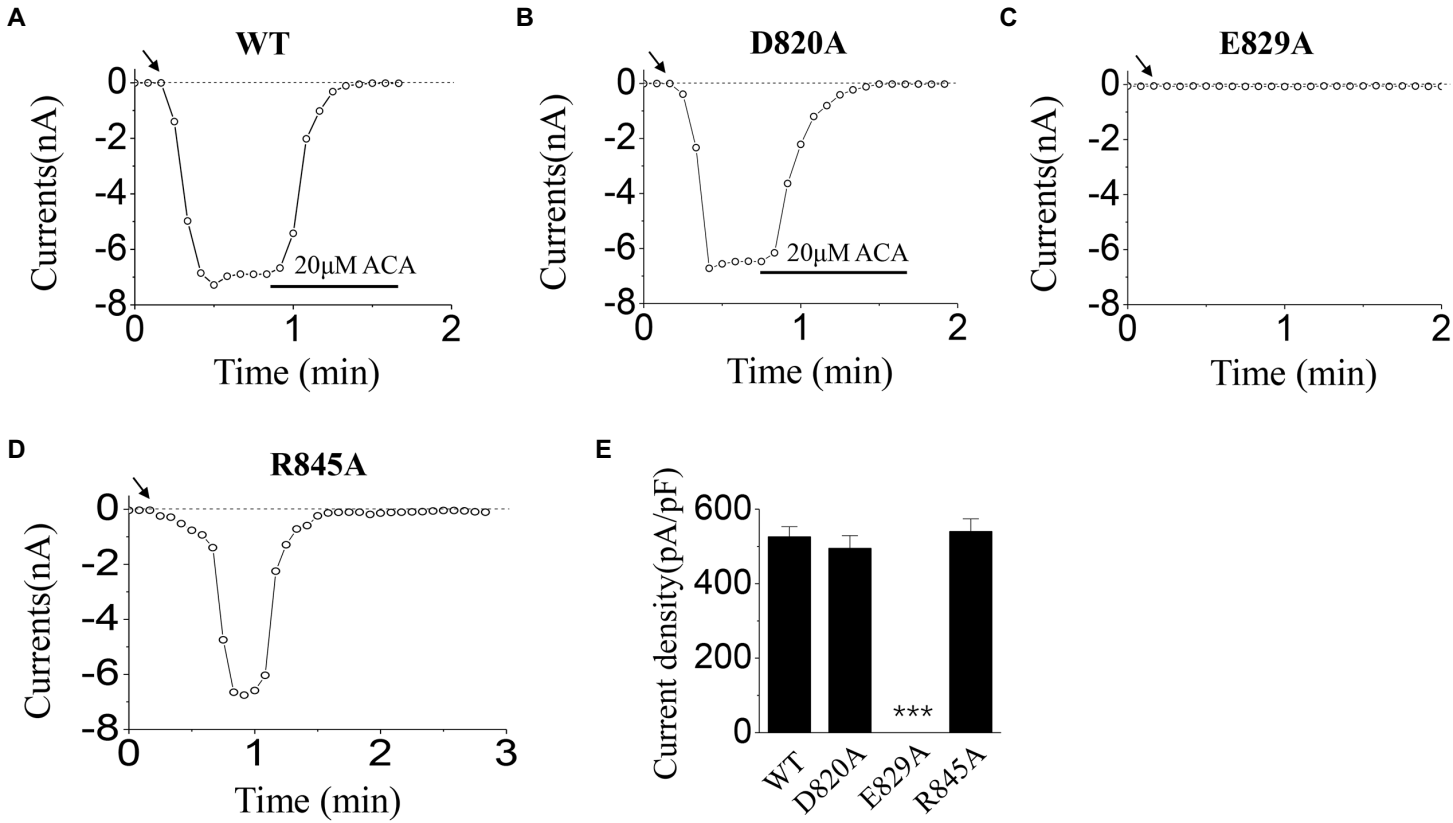
Functional assay of WT TRPM2 and its alanine mutants. **(A–D)** Representative whole-cell recordings of TRPM2 channel currents induced by ICS of 500 μM ADPR from HEK293T cells expressing **(A)** WT, **(B)** D820A, **(C)** E829A, and **(D)** R845A. The arrow in each panel indicates the time point at which whole cell configuration was established. **(E)** Summary of the current density induced in cells expressing WT and the mutants. In each case, six cells were tested. ^***^p < 0.001 compared with WT.

When using ICS of 500 μM ADPR, D820A behaved similarly with WT (Figures 3A,B,E): the currents of D820A were induced rapidly after the whole-cell configuration was established, and were inhibited completely by the channel inhibitor (20 μM ACA). However, E829A could not be activated by 500 μM ADPR (Figure 3C,E), and R845A exhibited inactivation which did not exist in WT TRPM2 (Figure 3D): After the peak value, the currents of R845A dropped rapidly until it entirely disappeared.

**Figure 4:**
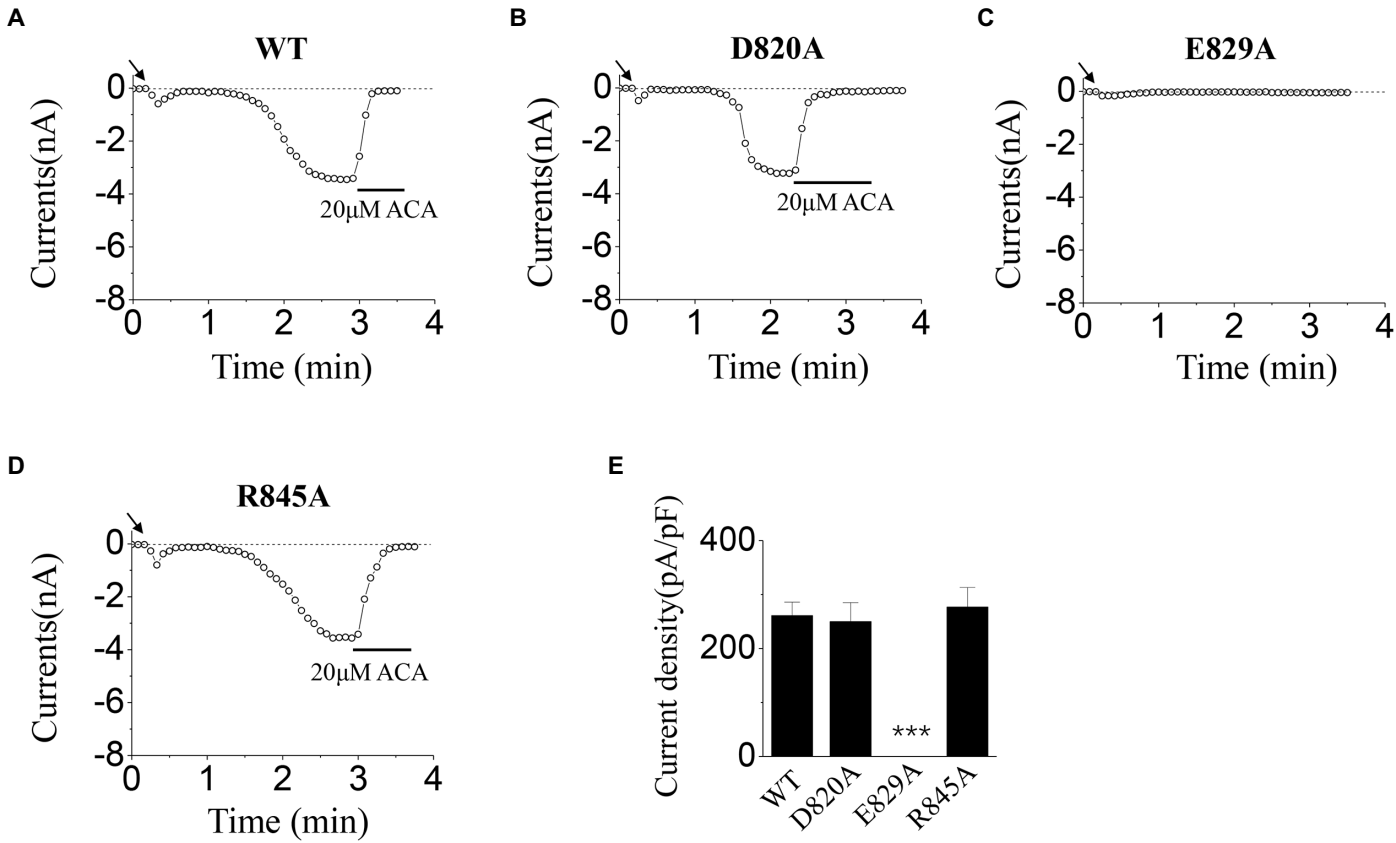
Functional assay of WT TRPM2 and its alanine mutants. **(A–D)** Representative whole-cell recordings of TRPM2 channel currents induced by ICS of 50 mM Ca^2+^ from HEK293T cells expressing **(A)** WT, **(B)** D820A, **(C)** E829A, and **(D)** R845A. The arrow in each panel indicates the time point at which whole cell configuration was established. **(E)** Summary of the current density induced in cells expressing WT and the mutants. In each case, six cells were tested. ^***^p < 0.001 compared with WT.

In the activation tests by ICS of 50 mM Ca^2+^, our results showed there was no significant difference between D820A, R845A, and WT (Figures 4A,B,D,E). However, no current was evoked for E829A by 50 mM Ca^2+^ (Figure 4C,E). All these data displayed the interesting phenomenon in the gating processes of mutants at E829 and R845, which needed our further exploration.

### The alternations of channel gating by mutations of E829K and R845D

**Figure 5:**
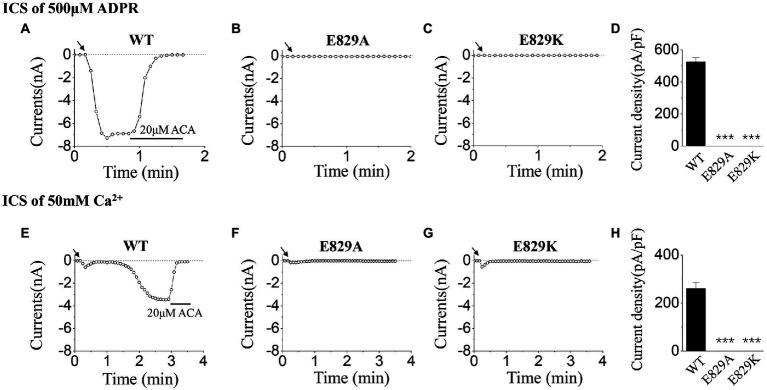
Functional assay of WT TRPM2 and mutants at E829. **(A–C)** Representative whole-cell recordings of TRPM2 channel currents induced by ICS of 500 μM ADPR from HEK293T cells expressing **(A)** WT, **(B)** E829A, **(C)** E829K, and **(D)** Summary of the current density induced in cells expressing WT and mutants. In each case, six cells were tested. **(E–H)** Representative whole-cell recordings of TRPM2 channel currents induced by ICS of 50 mM Ca^2+^ from HEK293T cells expressing **(E)** WT, **(F)** E829A, **(G)** E829K, and **(H)** Summary of the current density induced in cells expressing WT and mutants. The arrow in each panel indicates the time point at which whole cell configuration was established. In each case, six cells were tested. ***p < 0.001 compared with WT.

To explore the gating mechanism of E829, the positively charged mutant, E829K, which reserved the charge was designed, and the activation tests were done using ICS of 500 μM ADPR (Figures 5A–D) and 50 mM Ca^2+^ (Figures 5E–H). Together with the data of E829A, our whole-cell recordings presented the loss of function resulted from the mutations at E829: any alternation in charge at this residue (charge deletion mutant: E829A, or charge reversion mutant: E829K) led the channel failed to open.

**Figure 6:**
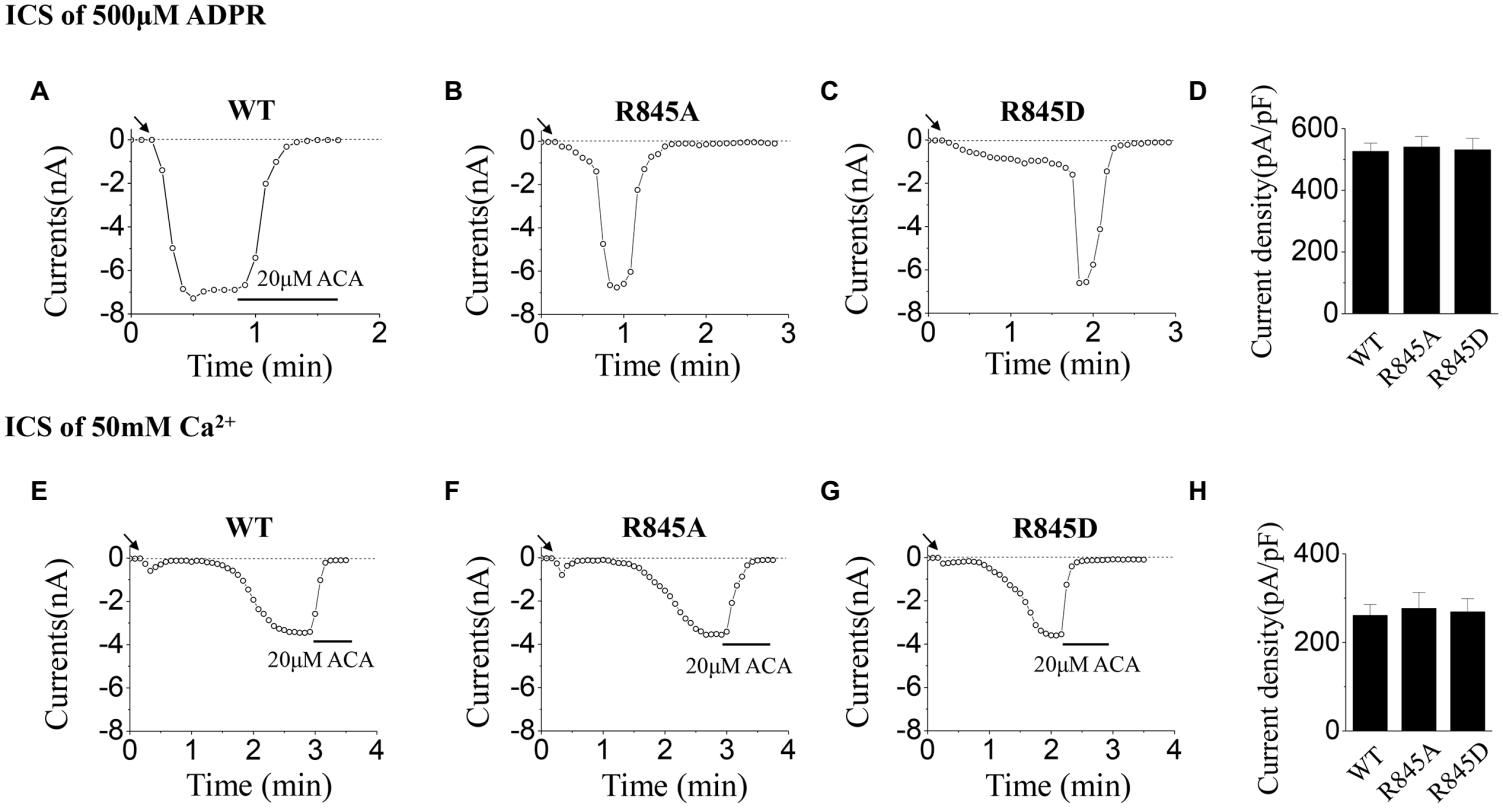
Functional assay of WT TRPM2 and mutants at R845. **(A–C)** Representative whole-cell recordings of TRPM2 channel currents induced by ICS of 500 μM ADPR from HEK293T cells expressing **(A)** WT, **(B)** R845A, **(C)** R845D, and **(D)** Summary of the current density induced in cells expressing WT and mutants. In each case, six cells were tested. **(E–H)** Representative whole-cell recordings of TRPM2 channel currents induced by ICS of 50 mM Ca^2+^ from HEK293T cells expressing **(E)** WT, **(F)** R845A, **(G)** R845D, and **(H)** Summary of the current density induced in cells expressing WT and mutants. The arrow in each panel indicates the time point at which whole cell configuration was established. In each case, six cells were tested.

As for R845, we constructed the negatively charged mutant, R845D, which also reversed the charge at this residue. During the activation tests by ICS of 500 μM ADPR, R845D behaved the characteristic of rapid inactivation as well as R845A (Figures 6A–D). On the contrary, no difference was seen between these two mutants and WT when activated by ICS of 50 mM Ca^2+^ (Figures 6E–H).

## Discussion

As a Ca^2+^-permeable channel that can be activated by Ca^2+^, Ca^2+^ binding at the S2-S3 domain of TRPM2 channel is a vital step for channel activation. After the channel opens, the Ca^2+^ influx facilitates the channel opening with positive feedback, and causes intracellular Ca^2+^ overload, which leads to cell death and neuronal loss (Jiang et al., 2010; Malko and Jiang, 2020). In this way, TRPM2 channel plays an important part in neurodegenerative diseases. Moreover, the upregulation of the TRPM2 channel was reported in human or animal with neurodegenerative diseases (For example, patients or mouse with Parkinson’s disease; Sun et al., 2018; Ferreira et al., 2022). All of these provide the foundations for TRPM2 channel as a potential biomarker in neurodegenerative diseases. Through the identification of the key residues in the D820-F867 segment around the S2 domain, our work promoted the understanding and development of TRPM2 channel. The results of electrophysiology showed that the residues E829 and R845 played an important part in channel gating. Mutations of E829A and E829K prevented the channel from being activated by 500 μM ADPR or 50 mM Ca^2+^, while R845A and R845D showed rapid inactivation when using ICS of 500 μM ADPR, but could be opened by 50 mM Ca^2+^.

Firstly, the activation tests were carried out for the three deletion mutants (△D820-W835, △L836-P851, and △D852-F867), using ICS of 500 μM ADPR. After the stability of the channel currents, 20 μM ACA was applied in the extracellular solution to exclude the data deviation caused by leakage currents. As our data showed, all of the currents induced could be inhibited totally (Figures 1A,D), suggesting that the currents we recorded resulted from the opening of TRPM2 channel. Considering the influence of cell size on the amplitude of the whole-cell currents, current density was used in statistical analysis. Among the three deletion mutants, △D852-F867 was the only one that reserved part of the currents with the deletion of 16 residues, indicating that the residues within D852-F867 segment have a relatively milder influence on channel gating (Figures 1D,E). On the contrary, deletion mutants of △D820-W835 and △L836-P851 resulted in a complete loss of channel function, indicating the existence of key residues in these two sequences (Figures 1B,C,E).

Based on the residue conservation and its polarity and charge, we focused on D820, E829, and R845 residues in △D820-W835 and △L836-P851 segments (Figure 2). The alanine mutants were constructed at these three residues with the eliminations of charge and side chain, and the activation tests by ICS of 500 μM ADPR or 50 mM Ca^2+^ were done to analyze the function of the mutants.

Our data displayed that the D820A mutant could be activated normally, and the characteristic of its currents was similar to WT TRPM2 (for example, the magnitude and stability), demonstrating the relatively small contribution of D820 during TRPM2 gating (Figures 3B, 4B). However, E829A made the channel lose function (Figures 3C, 4C), and R845A exhibited rapid inactivation in the activation test by ICS of 500 μM ADPR (Figure 3D), suggesting the involvement of these two residues in channel gating. To further explore the mechanism, we designed the charge-reversing mutants E829K and R845D, and detected their currents by electrophysiology.

As for mutants at E829, no current was induced by 500 μM ADPR or 50 mM Ca^2+^, neither E829A nor E829K (Figure 5). Former studies have illustrated that single site mutation did not affect TRPM2 channel expression, so the influences of channel gating by the mutations were focused (Luo et al., 2018, 2019). As a residue within the voltage-sensing-like domain of TRPM2 (Wang et al., 2018), the negative charge at E829 was involved in the series of conformational changes during channel opening. In this case, the alanine or lysine substitution which deleted or reversed its negative charge destroyed the linkage effect at this residue, thus resulting in the loss of function of the channel. Apart from mutation, the molecule combination at the channel voltage-sensing-like domain can also alter channel gating. For example, the structural changes caused by the inhibitor binding at the voltage-sensing-like domain of TRPC4 channel is the primary cause for channel closing (Vinayagam et al., 2020).

As for mutants at R845, the currents activated by ICS of 500 μM ADPR, exhibited fast inactivation after the peak currents, while the currents activated by ICS of 50 mM Ca^2+^ behaved similarly to WT (Figure 6). However, according to the structure of the TRPM2 channel, R845 is not within the ADPR binding site: MHR1/2 nor NUDT9-H domain, so the mutations here had little effect on the activation process by ADPR (Wang et al., 2018; Huang et al., 2019). Interestingly, R845 is located in the Ca^2+^ binding site of S2-S3 domain, just between E843 and Q846 (Wang et al., 2018). Considering the fact that Ca^2+^ binding in S2-S3 domain is the basis for the activation effect of ADPR, we proposed that R845 plays an important role in keeping the right construction of E843 and Q846 for Ca^2+^ binding, which guarantees the stability of Ca^2+^ binding here and also the opening of the channel. Any alternation of the charge at R845 will reduce the stability of Ca^2+^ binding, thus leading to inactivation. In the experiments using ICS of 500 μM ADPR, the concentration of Ca^2+^ is limited, during the opening of R845A or R845D, the change of the channel structure resulted in the dropping of Ca^2+^ from S2-S3 domain. However, there was not enough Ca^2+^ to fill this site, so the channel inactivated (Figures 6B,C). Only the WT TRPM2 channel, which bound Ca^2+^ tightly, displayed stable currents without any inactivation (Figure 6A). A similar phenomenon was also occurred in our former research about the effects of mutations at Q846 and N869 in TRPM2 gating process (Luo et al., 2019). Compared with R845A, the mutation of R845D made more changes to the charge at this residue, so it took R845D a much longer time to open (Figures 6B,C). On the other hand, in the experiments using ICS of 50 mM Ca^2+^, the high concentration of Ca^2+^ promised the binding of Ca^2+^ here, so R845A and R845D could be activated similarly to WT (Figures 6E–H).

In summary, our study identified two key residues: E829 and R845, in the D820-F867 segment around S2 domain of TRPM2. E829 participates in channel gating through its negative charge, and R845 affects channel gating through its influence on the stability of Ca^2+^ binding in the S2-S3 domain. Our work expanded the research about the mechanisms of the transmembrane region of TRPM2 in channel gating process, and provided a solid foundation for the study of the physiological and pathological functions of TRPM2 channel. The exploration of the mechanism of TRPM2 as a potential biomarker in neurodegenerative diseases also provides a new approach for the prediction, diagnosis, and prognosis of neurodegenerative diseases.

## Data availability statement

The datasets presented in this study can be found in online repositories. The names of the repository/repositories and accession number(s) can be found in the article/supplementary material.

## Author contributions

YL, CJ, and MF designed and performed the experiments and analyzed the data. YL conceived the study and wrote the manuscript. SC and FW revised the manuscript. All authors contributed to the article and approved the submitted version.

## Funding

This work was supported by the National Natural Science Foundation of China (No. 82272163 to MF) and the Joint-research Fund for Clinical Research of Affiliated Hangzhou First People’s Hospital (YYJJ2019Q08 to YL).

## Conflict of interest

The authors declare that the research was conducted in the absence of any commercial or financial relationships that could be construed as a potential conflict of interest.

## Publisher’s note

All claims expressed in this article are solely those of the authors and do not necessarily represent those of their affiliated organizations, or those of the publisher, the editors and the reviewers. Any product that may be evaluated in this article, or claim that may be made by its manufacturer, is not guaranteed or endorsed by the publisher.
